# Selenadiazole Inhibited Adenovirus-Induced Apoptosis through the Oxidative-Damage-Mediated Bcl-2/Stat 3/NF-κB Signaling Pathway

**DOI:** 10.3390/ph16101474

**Published:** 2023-10-16

**Authors:** Xia Liu, Jia Lai, Jingyao Su, Kelan Zhang, Jiali Li, Chuqing Li, Zhihui Ning, Chenyang Wang, Bing Zhu, Yinghua Li, Mingqi Zhao

**Affiliations:** Center Laboratory, Guangzhou Women and Children’s Medical Center, Guangzhou Medical University, Guangzhou 510120, China; liuxia@stu.gzhmu.edu.cn (X.L.); 2022210293@stu.gzhmu.edu.cn (J.L.); sujingyao@stu.gzhmu.edu.cn (J.S.); 18371090625@163.com (J.L.); lcq@stu.gzhmu.edu.cn (C.L.); ningzhihui@stu.gzhmu.edu.cn (Z.N.); wcy@stu.gzhmu.edu.cn (C.W.); zhubing@gzhmu.edu.cn (B.Z.)

**Keywords:** selenadiazole, adenovirus, ROS, apoptosis

## Abstract

Human adenovirus type 7 (HAdV7) infection causes severe pneumonia, yet there are still no breakthroughs in treatment options for adenovirus, and the road to antiviral drug development faces major challenges. We attempted to find new drugs and we stumbled upon one: selenadiazole. Selenadiazole has been shown to have significant anti-tumor effects due to its unique chemical structure and drug activity. However, its effectiveness against viruses has not been evaluated yet. In our study, selenadiazole also showed superior antiviral activity. In vitro experiments, selenadiazole was able to inhibit adenovirus-mediated mitochondrial-oxidative-damage-related apoptosis, and in in vivo experiments, selenadiazole was able to inhibit apoptosis by modulating the apoptotic signaling pathway Bcl-2/Stat3/NF-κB, etc., and was able to largely attenuate adenovirus-infection-induced pneumonia and lung injury in mice. This study aims to describe a new antiviral treatment option from the perspective of anti-adenovirus-mediated oxidative stress and its associated apoptosis and to provide theoretical guidance for the treatment of clinical adenovirus infection to a certain extent.

## 1. Introduction

Adenoviruses are nonenveloped viruses with an icosahedral capsid that measure between 70 and 90 nm in diameter. The AdV genome is composed of a linear double-stranded DNA molecule with reverse repeating sequences at both ends and an enclosed sequence. The length of the AdV genome ranges from 26 to 45 kilobase pairs (Kbp) [1]. The genomes of all adenoviruses are organized similarly, with early, mid, and late sections. Several early and late transcription units are encoded by the viral genome. The late region contains five transcript families, L1 to L5, that are crucial in the generation of mature virus particles [2]. These transcription units are differentially processed to produce a variety of mRNAs and proteins. Based on their biological, metabolic, genetic, and structural features, these serotypes are categorized into seven groups spanning from A to G. However, in other circumstances, particular serotypes might more severe infections, resulting in immunodeficient patients. They also pose a health risk to individuals with compromised immune systems [3]. AdV infection can cause a variety of clinical symptoms, including ophthalmic, respiratory, and gastrointestinal problems. Distinct serotypes of infection can also cause distinct diseases. Acute respiratory infections, for example, are caused by HAdV3, HAdV5, and HAdV7. AdV7 infections induce pneumonia in infants and recruits, while AdV21 infections have a clinical appearance comparable to bacterial pneumonia and AdV5 infections produce mild upper respiratory infections, with HAdV-B7 being of special concern [4]. Pneumonia, which can be deadly in children, and acute respiratory distress syndrome are two of the more significant complications of respiratory adenovirus infections [5].

Adenoviruses are a prevalent source of illness in both people and animals. Acute infections are particularly dangerous in youngsters and the immunocompromised. Adenoviruses attack several cell types, tissues, and organs in humans, and infections can be asymptomatic, mild, severe, or lethal. Some infections are severe, while others are chronic [6,7]. Human adenoviruses were originally isolated from cultured adenoid tissue, where they were able to persist in a dormant and symptom-free state [7]. They are subsequently associated with many clinical manifestations, including inflammatory conditions and non-inflammatory conditions [2]. Although adenovirus infection is usually asymptomatic in immunocompetent individuals, adenovirus disease has a substantial impact on children (particularly those under the age of four), the elderly, immunocompromised people, and recruits [8]. The immune system becomes active and produces a large number of chemokines and cytokines, which contribute to the onset of inflammation. This process is vital in the pathophysiology of tissue damage [9]. Cytokines, both chemotactic and non-chemotactic, have been proven to play a crucial role in host defense. Moreover, there is evidence that the cytokine response itself is crucial in the repercussions of AdV infection. Elevated IL-6, IL-8, and TNFα concentrations are related to the severity of AdV infection in children, including insufficient perfusion, peak temperature, and infectious shock [10].

Previously, the diagnosis of AdV infection was made by cultivating samples directly on MRC-5 lung fibroblasts or A549 lung adenocarcinoma cells, usually in conjunction with immunofluorescence detection of viral antigens [5,11]. With the ongoing progress in molecular detection techniques, the identification, categorization, and surveillance of adenovirus infection have gradually been incorporated into clinical routine, with tools to estimate the risk of invasive infection and provide a timely diagnosis [2,12]. The preferred diagnostic method for detecting AdV in peripheral blood samples is quantitative polymerase chain reaction (PCR). When compared to antigen detection methods (e.g., immunofluorescence) and culture, modern molecular approaches such as polymerase chain reaction (PCR) enable the rapid and sensitive identification of adenovirus nucleic acids [13]. After the AdV replication cycle, the viral genome can remain in the cell nucleus. The persistence of adenovirus after primary respiratory infection is best demonstrated by occasional viral excretion in nasopharyngeal secretions and feces [8]. However, treating AdV-related disorders in immunocompromised people remains a significant issue [14]. Despite the high prevalence and widespread use of AdV as a gene therapy vector and soluble tumor virus, specific FDA-approved anti-AdV therapies have not been available to date [3,15]. In contrast, broad-spectrum antivirals such as ribavirin, ganciclovir, and cidofovir, all of which prevent viral DNA replication, are used in clinical settings to treat severe adenovirus infections. Recently, brincidofovir has also been approved for use in this capacity [8,16]. Ganciclovir was initially used to treat herpesvirus infections, and there is little to no evidence that ribavirin can improve the course of adenovirus infections. However, similar to ribavirin, the majority of evidence points to ganciclovir’s limited efficacy as a HAdV treatment. Cidofovir is currently a standard of care. It was initially created to treat cytomegalovirus infection, although numerous in vitro studies and animal models have demonstrated that it is also an effective anti-adenoviral drug. Albeit as a highly recommended treatment, this medication has indisputable issues that significantly reduce its effectiveness, nephrotoxicity, and poor bioavailability [5,17].

Selenium (Se) has been well documented as an excellent trace element for humans and animals due to its wide range of pharmacological effects, important physiological functions, and essential components dependent on selenium enzymes [18]. Numerous studies from the last few decades have documented the use of selenium-containing species in biomedical research, including selenium nanoparticles, inorganic selenium, and organic selenium compounds. Researchers have shown a lot of interest in selenadiazole derivatives (SeDs), which are typical organic selenium compounds [19,20]. Selenadiazole has been proven to have some in vitro action against the DNA and RNA viruses represented in the test, despite the fact that its efficacy differs against various viruses [21]. During viral infection, selenadiazole’s impact on viral replication was strongest. In the current research, we verified that selenadiazole appears to have excellent in vitro antiviral activity. And even more, selenadiazole showed excellent in vivo antiviral activity in the presence of adenovirus infection through a selenium-deficient mouse model [22,23].

## 2. Results

### 2.1. Cytotoxicity and Antiviral Activity of Selenadiazole 

**Figure 1:**
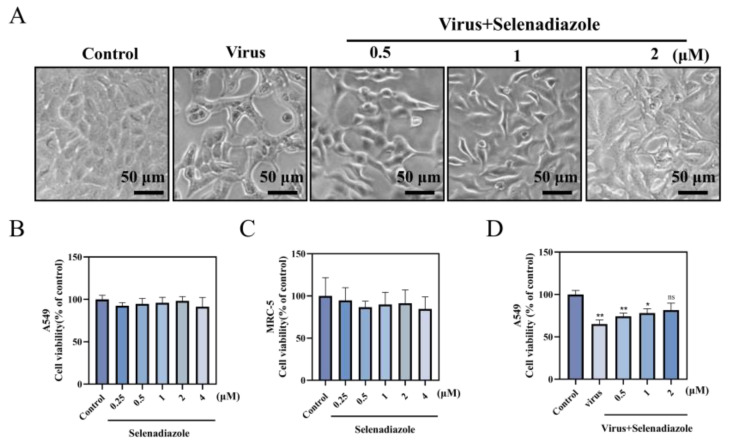
Morphological changes of A549 cells infected with adenovirus were observed by phase contrast microscopy. (**A**) The CCK8 assay was used to detect the effect of selenadiazole on the growth of A549 cells and MRC-5 cells infected with adenovirus type 7. Drug toxicity (**B**,**C**) and antiviral activity (**D**) of selenadiazole. The concentrations of selenadiazole were 0.5 μM, 1 μM, and 2 μM, and the bars of different characters were significantly different (ns *p* > 0.05, * *p* < 0.05 or ** *p* < 0.01).

To investigate the effect of selenadiazole on the proliferation of A549 cells infected with adenovirus, we employed the CCK-8 method to test the vitality of A549 cells treated with various concentrations of selenadiazole (0.5, 1, and 2 M) for 48 h [20,24]. Figure 1A demonstrates the in vitro effect of selenadiazole on adenovirus. Microscopical evidence of the drug’s overall in vitro effectiveness is presented, and an enzyme marker was used to determine the antiviral drug’s quantitative potency. Selenadiazole treatment had a significant effect on the morphology and adhesion of A549 cells compared to the control. Additionally, CCK-8 research demonstrated that selenadiazole significantly and dose-dependently reduced adenovirus-induced cell damage. Furthermore, selenadiazole’s inhibitory impact became more apparent with higher concentrations (Figure 1B,C) [25]. Regardless of the method of assessment, selenadiazole appears to be a very effective antiviral compound. In the study, selenadiazole showed the strongest inhibition of adenovirus at a concentration of 2 μM. The majority of antiviral medications have relatively little effect on adenovirus. However, in the current investigation, selenadiazole significantly inhibited AdV7 in A549 cells. Overall, the results suggest that selenadiazole has a concentration-dependent inhibitory effect on the production of apoptosis by virus infection of A549 cells. Meanwhile, we used normal human embryonic lung fibroblasts (MRC-5) to verify the cytotoxicity of selenadiazole. There was no obvious cytotoxicity in either A549 cells or MRC-5 cells.

### 2.2. Selenadiazole Inhibited Adenovirus-Induced Intracellular Reactive Oxygen Species Accumulation

**Figure 2:**
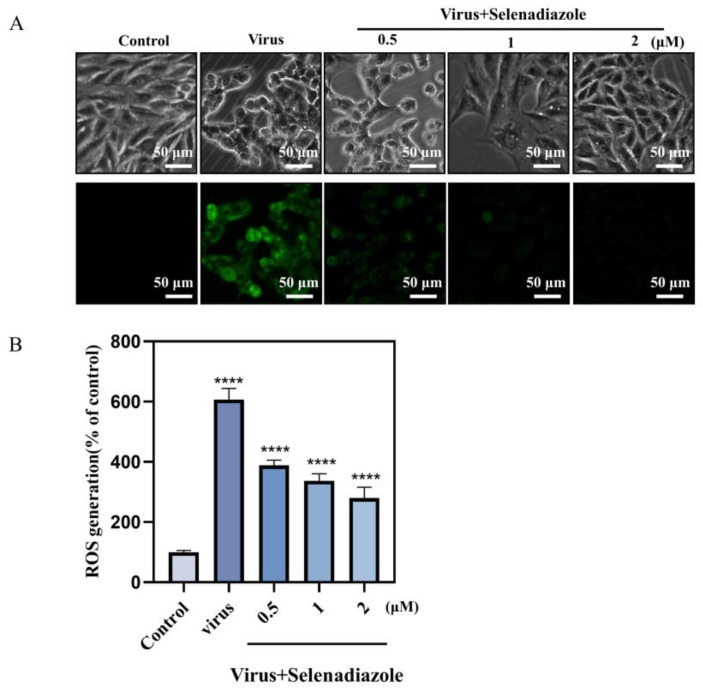
The inhibition of the overproduction of ROS by selenadiazole. ROS levels were detected by DCF fluorescence intensity. Adenovirus-infected A549 cells were treated with selenadiazole and then incubated with 10 mM DCF for 30 min. The concentrations of selenadiazole were 0.5 μM, 1 μM, and 2 μM. (**A**) Inverted microscope to observe the changes of A549 cells and their DCF fluorescence expres-sion intensity. (**B**) Enzyme marker to evaluate the intensity of DCF fluorescence produced by in-tracellular ROS oxidation. And the differences of bars with different characters were statistically significant (**** *p* < 0.0001).

We studied the impact of selenadiazole on apoptosis to determine whether the protective effect of the drug on cell growth was caused by inhibiting adenovirus-induced apoptosis. As expected, the proportion of apoptotic cells decreased dose-dependently after selenadiazole (0.5, 1, and 2 μM) treatment (Figure 2) [26,27].

### 2.3. Selenadiazole Inhibited Adenovirus-Induced Mitochondrial Membrane Potential Decline

**Figure 3:**
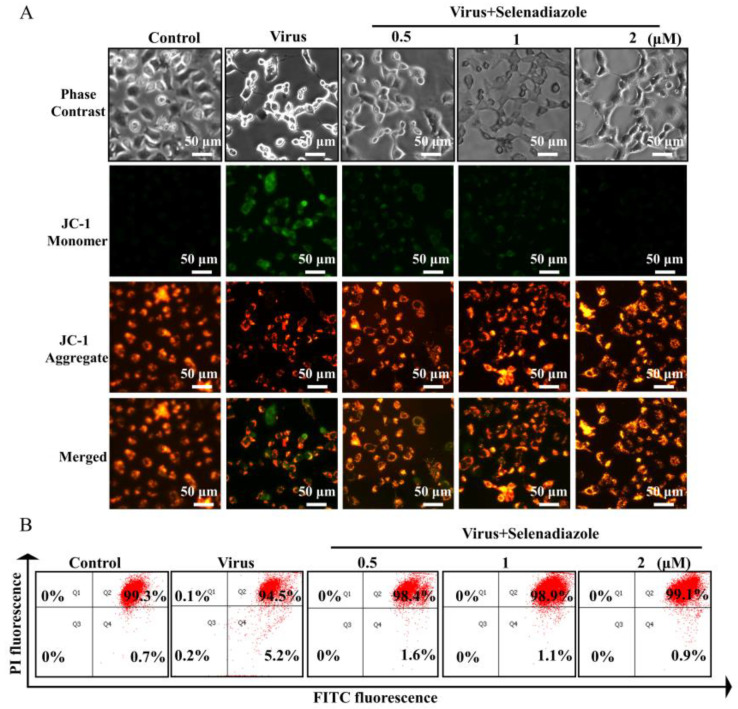
The detection of mitochondrial membrane potential in A549 cells. (**A**) JC-1 existed in the form of a polymer and shows bright red fluorescence in mitochondria, while the green fluorescence in cells was very weak. In A549 cells infected with adenovirus without selenadiazole treatment, JC-1 could not exist in the polymeric form in the mitochondrial matrix after the decrease of mitochondrial membrane potential. At this time, the red fluorescence intensity in mitochondria was significantly reduced, while the green fluorescence in the cytoplasm was significantly enhanced. (**B**) The results of the flow cytometry analysis.

Loss of mitochondrial membrane potential, a characteristic event of early apoptosis, can result from oxidative stress brought on by an increase in intracellular and mitochondrial ROS [28]. The shift of JC-1 fluorescence color can be utilized as a detecting signal of early apoptosis and is an easy way to identify a drop in membrane potential [29]. Next, in order to confirm our hypothesis, we performed JC-1 staining to detect the effect of selenadiazole on the mitochondrial membrane potential of the cells. Figure 3A shows that adenovirus-treated A549 cells showed increased mitochondrial depolarization and dysfunction, resulting in increased monomerization and reduced j-aggregates, which showed stronger green fluorescence. In contrast, selenadiazole treatment significantly reversed this phenomenon, showing a stronger red fluorescence [30]. Similarly, the cells could also be collected and examined using flow cytometry instrumentation, and as shown in Figure 3B, the proportion of early apoptotic cells was significantly reduced when A549 cells were exposed to selenadiazole compared to the adenovirus-infected group. The percentage of early apoptotic cells in the viral group was 5.2%, and the percentages of early apoptotic cells in the group that received selenadiazole were 1.6% (0.5 μM), 1.1% (1 μM), and 0.9% (2 μM) compared to the viral group. Thus, selenadiazole effectively prevented adenovirus-induced early apoptosis by reversing the decrease in mitochondrial membrane potential after adenovirus infection [31].

### 2.4. Selenadiazole Inhibited Adenovirus-Induced Apoptosis 

**Figure 4:**
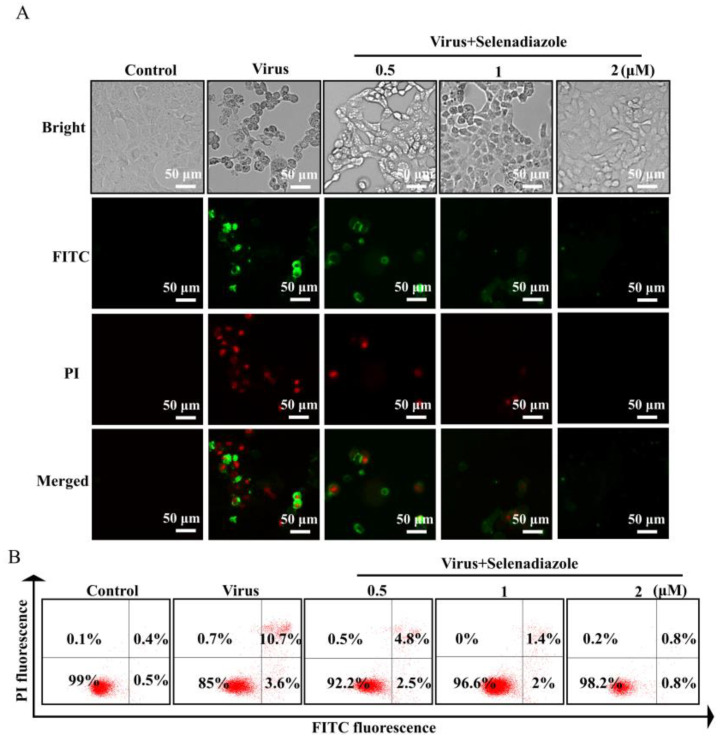
Annexin V-FITC and propidium iodide (PI) staining. (**A**) The green fluorescence is Annexin V-FITC-staining-positive cells, and the red fluorescence is propidium-iodide-staining-positive cells. Apoptotic cells were stained only by green fluorescence, necrotic cells were stained by both green and red fluorescence, and normal cells were not stained by fluorescence. Annexin V-FITC staining and propidium iodide staining were very weak in the selenadiazole treatment group, and A549 cells were in a normal state. The green fluorescence and red fluorescence were stronger in the virus treatment group alone. (**B**) The results of the flow cytometry analysis.

Annexin is a class of calcium-dependent phospholipid-binding proteins widely distributed in the cytoplasm of eukaryotic cells and is involved in intracellular signaling [32]. Annexin V selectively binds phosphatidylserine (PS). The inner side of the cell membrane and the side closest to the cytoplasm is where phosphatidylserine is most widely distributed. In the early stages of apoptosis, phosphatidylserine is transferred to the cell surface, the exterior of the cell membrane. The exposure of phosphatidylserine to the cell surface stimulates coagulation and inflammatory responses. Annexin V binds to the phosphatidylserine that has been ectopically transferred to the cell surface and blocks the pro-coagulant and pro-inflammatory activity of phosphatidylserine [33]. Annexin V-FITC is an Annexin V labelled with a green fluorescent FITC probe. It is possible to detect phosphatidylserine ectopics, an important feature of apoptosis, using flow cytometry or fluorescence microscopy [34]. As shown, both green and red fluorescence were strong in the virus-only-treated group, while fluorescence intensity diminished in a dose-dependent manner in the selenadiazole-treated group (Figure 4A). Flow cytometry analysis gave the same results as under the microscope. As can be seen from the figure, in the cells treated with selenadiazole (lower right quadrant of Figure 4B), the number of cells with positive Annexin V-FITC staining and negative PI staining, namely, apoptotic cells, was dramatically decreased as compared to the simple virus infection group. The cells with double-positive Annexin V-FITC and PI staining, namely, necrotic cells, were significantly decreased (upper right quadrant of Figure 4B). The cell spots in the quadrant (upper left quadrant of Figure 4B) of negative Annexin V-FITC staining and positive PI staining (Annexin V-/PI+) are the detection errors within the permissible limits [30,31,34]. 

### 2.5. Selenadiazole Inhibited the Inflammatory Response of Lung Tissue in Mice 

**Figure 5:**
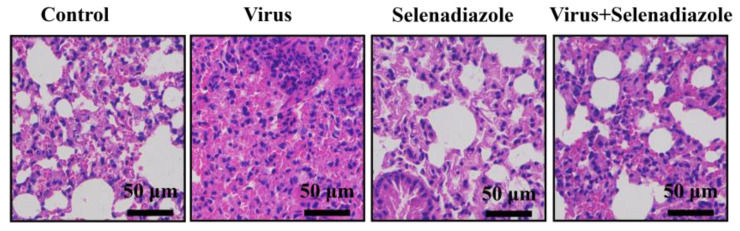
HE staining of mice lung tissue. Compared with virus infection group, selenadiazole treatment can effectively reduce the damage degree of alveolar structure in lung tissue of mice. Its shape is close to that of normal mice. In the group treated with selenadiazole, there was no obvious sign of lung injury and no obvious drug toxicity to mice.

To verify the antiviral effect of selenadiazole in vivo, we administered selenadiazole or a control agent to selenium-deficient mice. Selenadiazole treatment effectively reduced the destruction of alveolar structures in the lung tissues of mice with morphology close to that of normal mice compared to the virus-infected group alone, and there was no obvious sign of destruction in the lung tissues of mice treated with selenadiazole alone, indicating that selenadiazole had no pharmacologic effect on mice (Figure 5).

### 2.6. Selenadiazole Inhibited Adenovirus-Induced Apoptosis by Regulating Apoptosis-Pathway-Related Proteins

**Figure 6:**
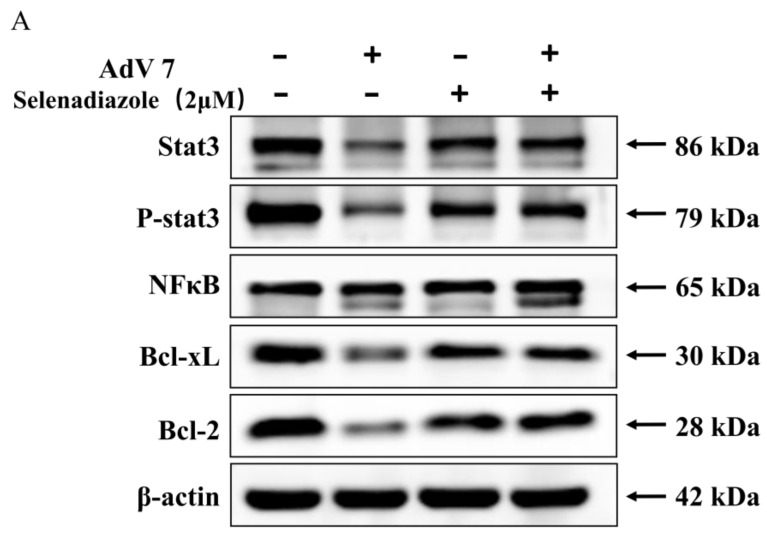

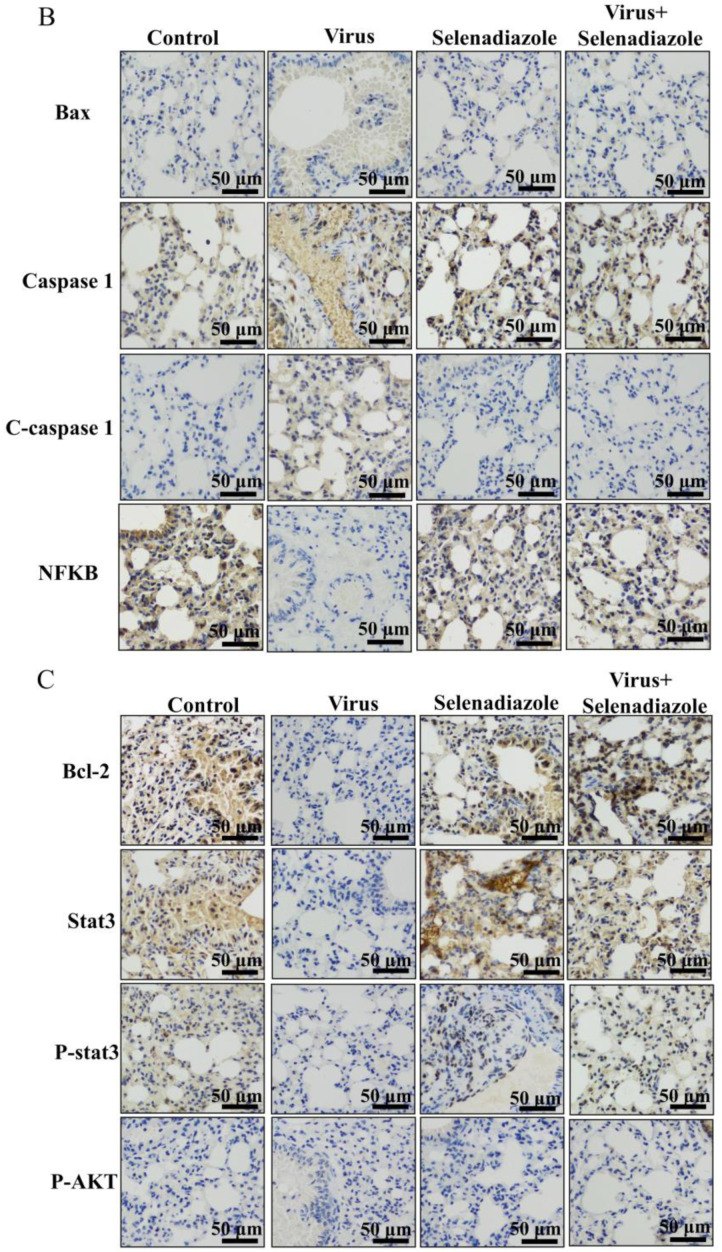
Selenadiazole reversed the damage of adenovirus infection to mouse lung tissue by upregulating the expression of proteins that inhibit apoptosis and downregulating the expression of proteins that promote apoptosis. (**A**) Intracellular apoptotic Stat3 and Bcl-2 signaling pathways by selenadiazole in HAdV infection of A549 cells. (**B**,**C**) The immunohistochemical analysis of mouse lung tissue. Selenadiazole treatment significantly increased the expression levels of Stat3, P-Stat3, Bcl-2, and NF-κB in mouse tissue sections and decreased the expression levels of Bax, caspase-1, cleaved caspase-1, and P-AKT.

Studies have shown that Stat3 is persistently activated in a variety of human tumors with oncogenic potential and has anti-apoptotic activity. In response to various apoptotic stimuli, Bcl-2 exerts a survival function by inhibiting mitochondrial cytochrome c release. It is implicated in the regulation of mitochondrial calcium homeostasis and proton flow [35]. As shown in Figure 6A, selenadiazole-treated cells significantly enhanced the expression levels of P-stat3 and stat3 in A549 cells compared to the viral group. Similarly, for the Bcl-2 signaling pathway, selenadiazole significantly increased the expression levels of Bcl-2 and Bcl-xL. These results reveal that selenadiazole inhibited adenovirus-induced apoptosis. The results of the immunohistochemical analysis were more meaningful. Selenadiazole treatment significantly increased the expression levels of Stat3, Bcl-2, and NF-κB and decreased the expression levels of Bax, caspase-1, cleaved caspase-1, P- Stat3, and P-AKT in mouse tissue sections (Figure 6). It was concluded that selenadiazole served to reverse the damage to mouse lung tissue caused by adenovirus infection by upregulating the expression of proteins that inhibit apoptosis and downregulating the expression of proteins that promote apoptosis [35,36,37].

### 2.7. Inhibition of Adenovirus Infection of Mice by Selenadiazole

**Figure 7:**
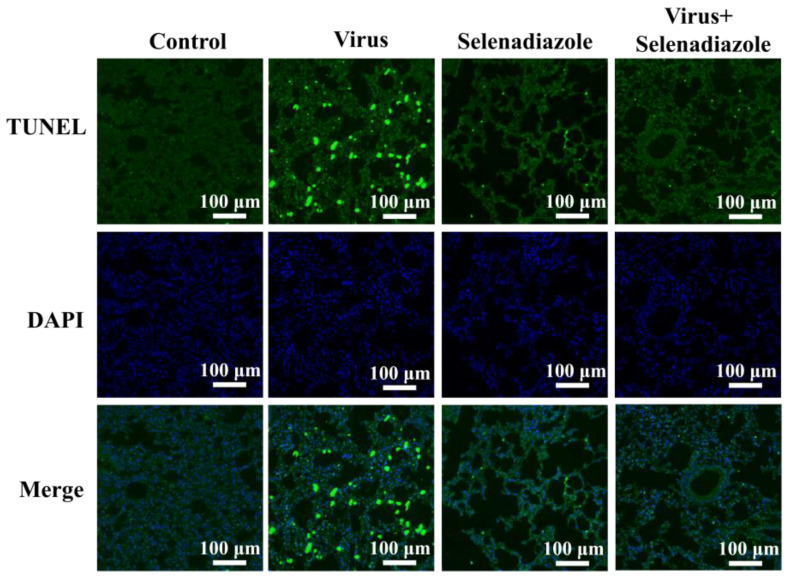
Selenadiazole reversed tissue damage induced by adenovirus infection in mice. The intensity of the green fluorescence in the figure, that is, the degree of nucleic acid breakage, reflects the level of apoptosis. The viral infection group had the strongest green fluorescence, indicating the most severe nucleic acid breakage, while the treatment of selenadiazole was similar to that of the normal group. The results indicated that selenadiazole inhibited adenovirus-mediated pneumonia by inhibiting apoptosis of lung tissue cells. All the results are representative of three independent experiments.

To verify the antiviral effect of selenadiazole in vivo, we administered selenadiazole or a control agent to selenium-deficient mice [38]. Selenadiazole treatment effectively reduced the destruction of alveolar structures in the lung tissues of mice with morphology close to that of normal mice compared to the virus-infected group alone, and there was no obvious sign of destruction in the lung tissues of mice treated with selenadiazole alone, indicating that selenadiazole had no pharmacologic effect on mice (Figure 7) [38,39,40].

### 2.8. Inhibition of Apoptosis Signaling Pathways by Selenadiazole

**Figure 8:**
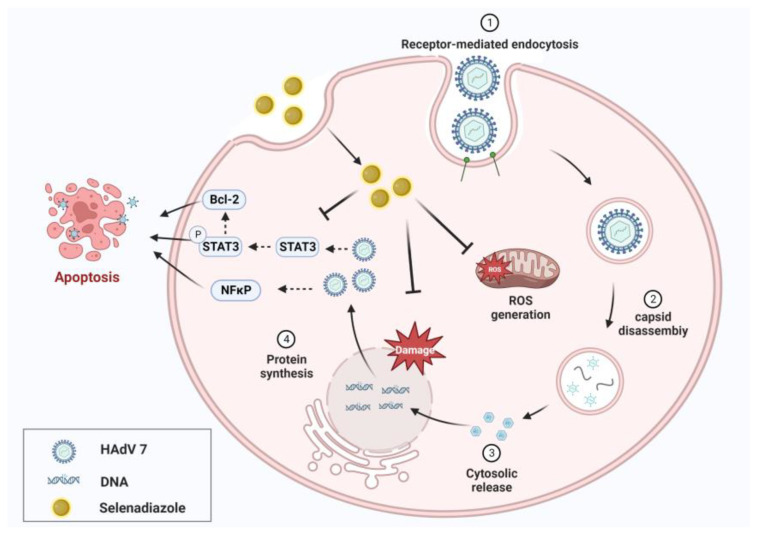
Inhibition of apoptosis signaling pathways by selenadiazole.

In general, adenovirus induced apoptosis of A549 cells through ROS-mediated signaling pathways, while selenadiazole was able to activate Bcl-2, Stat3, and NF-κB signaling pathways and inhibit apoptosis. This is shown in the Figure 8. These results suggest that Bcl-2, Stat3, and NF-κB play an important role in the treatment of adenovirus infection by selenadiazole.

## 3. Discussion

The predominance of selenium as an essential element in humans, its role in the initiation and development of numerous viral illnesses, its involvement in selenoproteins, and the level of selenium in host cells affects the replication of invading viruses, and although the antiviral effects of selenium and its derivative classes have been demonstrated in several studies, their antiviral mechanisms are not yet clear [21]. In our previous study, trace element selenium was found to have a certain antiviral effect. The inhibitory effect of selenium nanoparticles functionalized with oseltamivir on the H1N1 influenza virus was studied [41], and the inhibition of apoptosis induced by H1N1 influenza virus induced by β-thujaplicin surface-modified selenium nanoparticles was studied [42]. And the inhibitory effect of selenocysteine on HAdV-14-induced apoptosis was studied with good results [43]. In this study, we pioneered the use of a selenium derivative, selenadiazole, in the treatment of adenovirus infection in a model at the cellular and animal levels, and we provided a more comprehensive picture of the possible ways in which selenadiazole can be used to treat adenovirus, providing a promising and developmental advantage by inhibiting adenovirus-mediated apoptosis by inhibiting the replicative proliferative cycle of adenovirus and by regulating the intracellular apoptotic signaling pathway in response to adenovirus-mediated oxidative stress. 

It is generally recognized that elevated oxidative stress in cells is a major contributor to their malfunction and eventual demise. Reactive oxygen species (ROS) are produced in excess when adenoviruses infect cells, and mitochondria play a key role in this intracellular ROS generation. Overexpression of ROS impairs the production of ATP in the mitochondria, which results in mitochondrial malfunction and eventually apoptosis [26,27]. Intracellular reactive oxygen species (ROS) production is an important manifestation of apoptosis. This study aimed to test the hypothesis that selenadiazole could act as an inhibitor of oxidative stress, towards the apoptosis process in adenovirus-infected cells by regulating the expression of pro- and anti-apoptotic proteins. In the present work, the anti-adenoviral effect and inhibition of adenovirus replication of selenadiazole were confirmed by CCK-8, proving the accuracy of earlier reports. Subsequently, we showed whether the protective effect of selenadiazole on cell proliferation was mediated through blocking adenovirus-induced apoptosis by reactive oxygen species assay. We also performed JC-1 staining to examine the effect of selenadiazole on cell mitochondrial membrane potential and showed that selenadiazole effectively prevented adenovirus-induced early apoptosis by reversing the decrease in cell mitochondrial membrane potential after adenovirus infection. Annexin-V/PI double staining confirmed that adenovirus induced apoptosis and selenadiazole inhibits apoptosis.

Further exploring its antiviral mechanism, it was revealed that selenadiazole treatment effectively attenuated the destruction of alveolar structures in mouse lung tissues. In addition, immunohistochemical analysis of mouse lung tissues showed that selenadiazole reversed the damage caused by adenovirus infection to mouse lung tissues by upregulating the expression of apoptosis-inhibiting proteins and downregulating the expression of apoptosis-promoting proteins. In order to identify cellular DNA damage in lung tissue after viral infection, TUNEL-DAPI labeling was employed. Following viral infection, selenadiazole was observed to prevent DNA damage in lung tissue cells. This was attributable to the fact that selenadiazole reduced the accumulation of virus-mediated ROS and thus prevented ROS damage.

## 4. Materials and Methods

### 4.1. Materials

Two continuous cell lines were used in this study: the A549 cell line and the MRC-5 cell line. Cells were grown in minimum essential medium (DMEM) supplemented with 10% fetal bovine serum (FBS; KC Biologics, Lenexa, KS, USA) and 1% penicillin and streptomycin, and A549 cells were grown in a mixture of DMEM supplemented with 10% fetal bovine serum and 1% penicillin and streptomycin. All cells were passaged in 25 cm^3^ or 75 cm^3^ plastic flasks (Corning Glass Works, Corning, NY, USA) at 37 °C with 5% CO_2_ humidified by air. The experiments performed in this paper were all carried out in our laboratory with cells that were passaged 5~30 times. Regular tests revealed that no cell cultures contained any bacterial or mycoplasma contamination. All animal experiments in this study were approved by the Experimental Animal Ethics Committee of Guangzhou Medical University. Male C57BL/6 mice that were 8 weeks old and gland free were purchased from CLEA Japan (Tokyo, Japan) and were fed sterile chow and water while being kept pathogen free. The mice were randomly assigned to 4 groups (3 mice in each group) and anesthetized with 10% chloral hydrate 3 μL/g. The control group and drug group were treated with 20 μL saline nasal drops. The virus group and virus + drug group were treated with 20 μL adenovirus nasal drops. After 24 h, selenadiazole was administered to anesthetized mice by intranasal absorption three times every 24 h. Mice were weighed every week until the last day [24], and the lung tissue samples of mice were perfused with normal saline, fixed with paraformaldehyde, stained with hematoxylin and eosin (H&E) for histopathological evaluation, and paraffin sections were prepared for immunohistochemical studies.

### 4.2. Viral Pools Were Prepared by Infecting Fused A549 Monolayers

The AdV 7 strain involved in this experiment was derived from respiratory tract and pharyngeal swab samples of patients with positive AdV 7 infection during the onset of the disease, being isolated and cultured in the central laboratory of Guangzhou Women and Children’s Medical Center, affiliated with Guangzhou Medical University. Infected monolayers were cultivated at 37 °C, 5% CO_2_ for 2–4 days before being collected when the cultures had 90% of their cytopathic effect (CPE). Viruses were passaged at least twice in this cell line before the use of the virus pool. Infected cells and fluids are frozen at −20 °C. The intracellular virus was released by freeze–thawing and violently squeezing the infected cells at the flask wall to destroy them, repeatedly freeze–thawing three times to finally harvest the viral fluid. The virus medium was divided into 0.3 mL samples, frozen, and stored at PA −80 °C until use. The Reed and Muench method was used to test adenovirus samples for infectious viruses in order to calculate the 50% tissue culture infectious dosage.

### 4.3. Antiviral Compound Preparation

The selenadiazole used in this study was provided by the Institute of Chemistry and Materials, Jinan University.

### 4.4. Cytotoxic and Antiviral Effects of Drugs

A549 cells were used for normal cytotoxicity studies. These cells were inoculated onto 96-well plates (Corning) at a concentration of 0.1 mL of 8 × 10^4^ cells per well and incubated at 37 °C in 5% CO_2_ for 24 h until near fusion. They were divided into 4 groups: normal cell group, drug-treated group alone, virus-treated group alone, virus and drug-treated group. A specified 50% tissue culture infection dosage of the virus was used to infect the cells on the plates (0.1 mL per well), and the medium was then changed to a growth medium containing various doses of selenadiazole. For each drug concentration, three sets of replicate wells were employed, along with an equal number of control wells (normal cell group, drug-treated group, and virus-treated group). The cells were exposed to the drug for 24–48 h, after which the cells’ proliferation was viewed under a microscope. Later, using the CCK-8 technique, the impact of selenadiazole on the proliferation of A549 cells was evaluated. The cells in 96-well plates were taken out of the medium and given a PBS wash, and then the CCK-8 solution was added. The cells were then incubated at 37 °C for 1 h. The absorbance of live cells was later measured at 450 nm using an enzyme marker.

### 4.5. Intracellular Reactive Oxygen Species Accumulation Was Detected Using a DCFA-DA Probe 

Overexpression of ROS impairs ATP synthesis in the mitochondria and results in dysfunctional mitochondria, which then triggers apoptosis. In the physiological process of apoptosis, ROS is essential. In order to identify reactive oxygen species, we injected the cells in 12-well plates, infected them with the virus by the earlier experimental methods, and then utilized the fluorescent probe DCFH-DA (2,7-dichlorodihydrouridine diacetate). DCFH-DA can easily pass through the cell membrane while not producing any light of its own. When DCFH-DA enters the cell, it can be digested by intracellular esterases to generate DCFH. The probe can be easily put into the cell because DCFH does not penetrate the cell membrane. Non-fluorescent DCFH can be oxidized by intracellular reactive oxygen species to yield luminous DCF. The fluorescence of DCF can be used to estimate the level of intracellular reactive oxygen species. First, we prepared a diluted solution of DCFH-DA in a medium without serum, using a ratio of 1 part DCFH-DA to 1000 parts medium. Then, we replaced the original cell medium with an appropriate amount of the diluted DCFH-DA solution. Following this, for 30 min, it was incubated in a cell culture chamber at 37 °C. Cells were washed three times with serum-free medium to completely remove the DCFH-DA that had not entered the cells and the cells were collected. Mitochondrial ROS levels were then obtained by fluorescence intensity analysis of DCF at 488 nm excitation and 525 nm emission using an enzyme marker.

### 4.6. Measurement of Mitochondrial Membrane Potential by JC-1 Fluorescence 

By quickly and sensitively identifying changes in mitochondrial membrane potential in cells or tissues, JC-1 can be employed for the early diagnosis of apoptosis. When JC-1 is a polymer (J-aggregate) at high mitochondrial membrane potentials, it can produce red fluorescence in the mitochondrial matrix (matrix). When JC-1 is a monomer at low mitochondrial membrane potentials, it cannot produce red fluorescence in the mitochondrial matrix but can instead produce green fluorescence. In A549 cells subjected to selenadiazole as previously described, the fluorescence intensity of JC-1 was utilized to determine the status of ΔΨm, and selenadiazole-treated A549 cells showed modifications in their plasma membrane. As stated above in the experimental steps, we injected the cells on 6-well plates and infected them with the virus. The original culture medium in the six-well plate was aspirated; the cells were washed once with PBS; and 1 ml of cell culture medium, which can contain serum and phenol red, was added. Subsequently, 1 mL of JC-1 staining working solution was added and properly combined. The mixture was incubated for 20 min at 37 °C in a cell incubator. The supernatant was aspirated and twice rinsed with JC-1 staining buffer (1X) following the incubation at 37 °C. We used fluorescence microscopy or gathered cells for a flow cytometric study after adding 2 mL of cell growth media.

### 4.7. Detection of Membrane Phosphatidylserine Turnover and Permeability Changes in Cells 

The FITC-labeled recombinant human Annexin V was used in the Annexin V-FITC Apoptosis Detection Kit to identify phosphatidylserine on the cell membrane during apoptosis. The kit also includes a solution for propidium iodide (PI) staining, which stains necrotic cells or cells that have lost the integrity of their cell membranes late in the apoptotic process with red fluorescence. Because the integrity of the cell membrane has been compromised, Annexin V-FITC can penetrate necrotic cells’ plasma and bind to phosphatidylserine on the inner side of the cell membrane, resulting in green fluorescence in these cells as well. As instructed by the kit, the original cell culture was aspirated into a suitable centrifuge tube, the walled cells were washed once with PBS, and the cells were digested by adding an appropriate amount of trypticase cell digest (as free of EDTA as possible). The cells were also inoculated on a 6-well plate and infected with the virus as previously described. The cells are gently blown down and transferred to a centrifuge tube. The supernatant was discarded, and 195µL of Annexin V-FITC conjugate was added to gently resuspend the cells. We added 5µL Annexin V-FITC and 10 µL propidium iodide staining solution, mixed them gently, and incubated the mixture for 10–20 min at room temperature (20–25 °C), protected from light, for flow cytometric analysis. It was not necessary to digest the cells down if they were observed by fluorescence microscopy.

### 4.8. HE Staining of Mice Lung Tissue

We examined mouse lung tissue using HE staining to see how selenadiazole affected the level of damage to lung tissue in adenovirus-infected mice. Cells were treated for 24 h with varied doses of selenadiazole. Tissues were fixed in 4% PFA-PBS for 12 h at 4 °C for paraffin sections. They were then dehydrated with ethanol and xylene, embedded in paraffin, and serially sectioned (4 μm in thickness). Histological staining (HE staining) was performed on all sections.

### 4.9. Western Blot Analysis and Immunohistochemical Staining of Mice Lung Tissue

The expression levels of apoptosis-related proteins were detected by Western blot. Immunohistochemical analysis was used to assess the expression levels of Stat3, Bcl-2, NF-κB, Bax, caspase-1, cleaved caspase-1, P-Stat3, and P-AKT in mouse lung tissue using a previously reported methodology. Briefly, 4 mm serial sections were dewaxed in xylene, rehydrated in a graded ethanol series, and immersed in EDTA antigen extraction buffer in a microwave oven for 15 min. To inhibit endogenous peroxidase activity, sections were treated for 20 min with 3% hydrogen peroxide in anhydrous methanol. Then, for 15 min, 5% bovine serum albumin was administered to prevent non-specific binding. Overnight at 4 °C, sections were incubated with primary antibody. Abcam (Cambridge, UK) provided Stat3, Bcl-2, NF-κB, Bax, caspase-1, cleaved caspase-1, P-Stat3, and P-AKT. Following secondary antibody incubation, the signals were developed using 3,3’-Diaminobenzidine (DAB) Tetrahydrochloride.

### 4.10. TUNEL-DAPI Staining of Mice Lung Tissue

Mouse lung tissue was sectioned by terminal deoxynucleoside transfer-mediated incision end labeling (TUNEL), and the slides were examined under a light microscope. When apoptosis occurs, several DNA endonucleases are activated, which sever genomic DNA between nucleosomes. The 180–200 bp of DNA ladder can be found when DNA is extracted for electrophoresis during apoptosis. When genomic DNA is broken, the exposed 3’-OH can be catalyzed by terminal deoxynucleotidyl transferase (TdT) plus the green fluorescent probe fluorescein (FITC)-labeled dUTP (fluorescein-dUTP), allowing observation by fluorescence microscopy. Mouse lung tissue was taken, frozen sections were made, and the cells were then fixed in immunostaining fixative (P0098) or 4% paraformaldehyde produced by Beyoncé for 30–60 min. Immunostaining Power Permeabilisation Solution (P0097) or PBS containing 0.5% Triton X-100, produced by Beyoncé, was then added and incubated for 5 min at room temperature. After 2 washes with PBS, the samples were evenly covered with the prepared TUNEL assay solution and incubated for 60 min at 37 °C, protected from light. Then, they were observed by fluorescence microscopy.

### 4.11. Statistical Analysis

Every experiment was carried out at least three times. The data are given as mean ± standard deviation. The difference between groups was examined using variance analysis, and the two-tailed test was performed using SPSS26.0 software. *p* < 0.05 (*) was deemed statistically significant.

## 5. Conclusions

In conclusion, selenadiazole was not significantly cytotoxic in this investigation and had a high antiviral potential to limit adenovirus infection. Its antiviral mechanism suggested that selenadiazole mediated apoptosis of A549 cells through upregulating Bcl-2/Stat3/NF-κB, and in addition, selenadiazole showed satisfactory antiviral effects in vitro. In particular, selenium is a crucial nutrient that is found in trace amounts in various healthy tissues throughout the body, and low concentrations of selenium derivatives even help to provide anticancer and antioxidant effects. Therefore, the current study suggests that selenadiazole can be widely used in functional studies of antiviral drugs, whether for administration or other therapeutic purposes, and the utilization of selenadiazole strategies has the potential to be an extremely effective approach in managing adenovirus transmission, a crucial step towards the creation of novel antiviral medications.

## Data Availability

The datasets supporting the conclusions of this article (are) included within the article.
